# TAM receptors Tyro3 and Mer as novel targets in colorectal cancer

**DOI:** 10.18632/oncotarget.10889

**Published:** 2016-07-28

**Authors:** Robin Schmitz, Aida Freire Valls, Rosario Yerbes, Sophie von Richter, Christoph Kahlert, Sonja Loges, Jürgen Weitz, Martin Schneider, Carmen Ruiz de Almodovar, Alexis Ulrich, Thomas Schmidt

**Affiliations:** ^1^ Department of General, Visceral and Transplantation Surgery, University Hospital Heidelberg, University Heidelberg, Heidelberg, Germany; ^2^ Biochemistry Center, University of Heidelberg, Heidelberg, Germany; ^3^ Department of Visceral, Thoracic and Vascular Surgery, University of Dresden, Dresden, Germany; ^4^ Department of Tumor Biology, Center of Experimental Medicine, University Medical Center Hamburg-Eppendorf, Hamburg, Germany

**Keywords:** colorectal cancer, tyro3, Mer, Gas6, macrophages

## Abstract

**Purpose:**

CRC remains the third most common cancer worldwide with a high 5-year mortality rate in advanced cases. Combined with chemotherapy, targeted therapy is an additional treatment option. However as CRC still escapes targeted therapy the vigorous search for new targets is warranted to increase patients' overall survival.

**Results:**

In this study we describe a new role for Gas6/protein S-TAM receptor interaction in CRC. Gas6, expressed by tumor-infiltrating M2-like macrophages, enhances malignant properties of tumor cells including proliferation, invasion and colony formation. Upon chemotherapy macrophages increase Gas6 synthesis, which significantly attenuates the cytotoxic effect of 5-FU chemotherapy on tumor cells. The anti-coagulant protein S has similar effects as Gas6.

In CRC patient samples Tyro3 was overexpressed within the tumor. *In-vitro* inhibition of Tyro3 and Mer reduces tumor cell proliferation and sensitizes tumor cells to chemotherapy. Moreover high expression of Tyro3 and Mer in tumor tissue significantly shortens CRC patients' survival.

**Experimental design:**

Various *in vitro* models were used to investigate the role of Gas6 and its TAM receptors in human CRC cells, by stimulation (rhGas6) and knockdown (siRNA) of Axl, Tyro3 and Mer. In terms of a translational research, we additionally performed an expression analysis in human CRC tissue and analyzed the medical record of these patients.

**Conclusions:**

Tyro3 and Mer represent novel therapeutic targets in CRC and warrant further preclinical and clinical investigation in the future.

## INTRODUCTION

Colorectal cancer (CRC) remains the third most common cancer worldwide with an incidence of nearly 1 million cases per year [1] and approximately half a million cancer related deaths per year [1, 2]. Current treatment of CRC contemplates adjuvant chemotherapy after surgery in more advanced cases, or chemotherapy together with targeted therapy in metastatic CRC [1, 2]. The 5-year survival of patients depends on the tumor stage and regional medical standard of patient care with an average overall 5-year survival of 60% in the US [3]. Even though the progression of colorectal cancer is increasingly understood we are still struggling to identify further targets to improve patients' outcome.

The TAM receptor family was discovered as one of the most recent tyrosine kinase receptor families [4, 5]. When discovered in 1991 the receptors were considered as orphan receptors and were in great parts identified as oncogenic drivers in hematologic diseases [6]. Only in 1995 the two ligands Gas6 and protein S (Pros1) were identified as binding ligands for the TAM receptors [7, 8]. Gas6 was discovered in growth arrested fibroblasts (therefore named Growth arrest-specific gene 6) and exerts pleiotropic functions in health and disease [6]. Gas6 is known to amplify platelet aggregation and thrombus formation [9, 10], enhances erythropoiesis and increases leucocyte extravasation in inflammation [11, 12]. While Gas6 binds to all TAM receptors protein S only activates Tyro3 and Mer [6]. Both TAM receptors and Gas6 have been shown to be overexpressed in a variety of solid cancers and especially hematological tumors. Frequently a correlation with poor prognosis and advanced tumor stage is found [5].

The role of Gas6/TAM receptors in solid tumors is still not completely elucidated. Gas6 expression was shown in tumor cells of gastric cancer, ovarian cancer and glioblastoma multiforme (GBM) [13–15], unlike renal cell carcinoma and breast cancer [16–18]. Furthermore Gas6 may also be expressed in tumor-associated cells like macrophages [19], which we have previously shown in animal models [20]. Although Gas6 expression was shown to be usually higher in tumor tissue, a correlation to survival was not confirmed throughout different tumor entities [6]. The Axl receptor is similarly overexpressed within the tumor tissue of different cancers (i.e. breast cancer, renal cell carcinoma, GBM, ovarian cancer, pancreatic cancer and esophageal cancer), and high Axl expression was associated with a shorter survival and more advanced tumor stage [6].

Less is known about the role of Tyro3 and Mer in human cancers. Mer plays a role in multiple myeloma and acute lymphoblastic leukemia [21–23], it was promoted as a novel therapeutic target in GBM [24] and co-expression with Axl correlated with worse survival in gastric cancer [25]. Tyro3 is even less studied. It was recently indicated that Tyro3 expression increases survival of malignant melanoma cells [26]. Moreover, it plays a role in a Tyro3/Axl autocrine signaling circuit to sustain malignancy in thyroid carcinoma [27] and is important for proliferation in breast cancer [28].

Whereas the role of protein S in the coagulation system is well known, hardly anything is known about its function as a ligand for the TAM receptors in cancer, while some studies indicate an (over) expression of protein S in different cancers [29]. In summary the role of Gas6/protein S and the TAM receptors remains controversial in solid tumors with different roles depending on the tumor entities. Especially the current available data in CRC is inconclusive.

Our current study intends to overcome the lack of knowledge on the role of the TAM receptors and their ligands in CRC and to shed more light on the currently available controversial data. We aim to comprehensively study the expression of Gas6, Protein S and the individual TAM receptors in a large CRC patients cohort and to dissect the influence of each receptor.

## RESULTS

### Enhanced expression of Gas6 and its TAM receptor Tyro3 in human colorectal cancer samples

**Figure 1:**
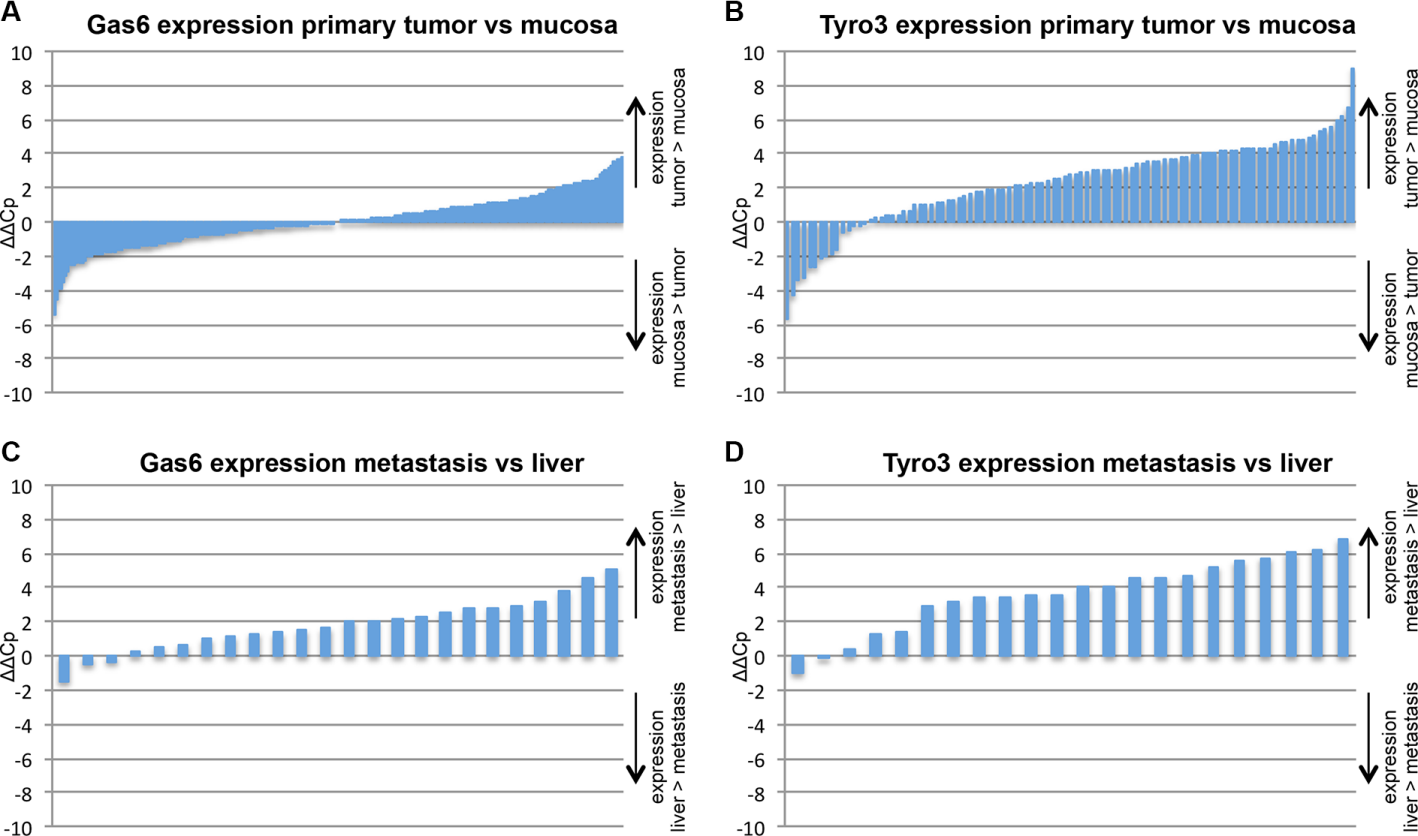
mRNA expression analysis in human CRC tissue Each bar represents one patient. Positive bars represent higher expression in tumor (**A**, **B**) or metastasis (**C**, **D**) compared to normal mucosa or normal liver tissue. Negative bars represent higher expression in mucosa or normal liver tissue compared to tumor or liver metastasis. Relative Gas6 (A) or Tyro (B) mRNA expression in human CRC tumor samples vs normal mucosa of the same patients respectively (*n* = 200; *P* = N; and *n* = 103; *P* < .05). Gas6 (C) and Tyro3 (D) mRNA expression in human CRC liver metastases and normal liver tissue of the same patients respectively (*n* = 24; *P* < 0.05; *n* = 24; *P* < 0.05).

To assess if the ligand Gas6 or its TAM receptors are differentially expressed within tumors in comparison to normal intestinal mucosa of the same patients, we performed qPCRs from 200 primary tumors with different tumor stages (UICC stage I–IV). Patients' characteristics are listed in Table 1A. Gas6, Axl and Mer were differentially expressed in tumors, with neither a general higher nor lower expression (Figure 1A and Supplementary Figure S1A, S1B). However, Tyro3 was overexpressed within the tumor in comparison to normal colon mucosa (*p* < 0.0001) (Figure 1B).

**Table 1:** Patient characteristics for mRNA expression analysis of Gas6, Axl, Mer, Tyro3 and ProteinS

(A) Summary of patient characteristics used for mRNA expression analysis of the target genes Gas6, Axl, Mer, Tyro3 and ProteinS in colorectal cancer tissue and normal mucosa of each patient respectively (n = 200)	
	*n* (%) or median (IQR)
Total *n*	200 (100)
**Gender**	
male	114 (57)
female	86 (43)
**Age (years)**	65,13 (27–88)
**Tumor size**	
T1	10 (5)
T2	46 (23)
T3	119 (59,5)
T4	25 (12,5)
**Lymph node status**	
positive	99 (49,5)
negative	101 (50,5)
**Distant metastases**	
positive	55 (27,5)
negative	145 (72,5)
**UICC**	
I	41 (20,5)
II	51 (25,5)
III	55 (27,5)
IV	53 (26,5)
**Tumor differentiation**	
high (G1)	1 (0,5)
moderate (G2)	153 (76,5)
poor (G3)	44 (22)
N.N.	2 (1)
**Tumor location**	
colon	101 (50,5)
rectosigmoid	13 (6,5)
rectum	86 (43)
**Neoadjuvant therapy**	
Yes	46 (23)
No	151 (75,5)
N.N.	3 (1,5)
**Adjuvant therapy**	
Yes	99 (49,5)
No	101 (50,5)
**Treatment**	
curative (R0)	155 (77,5)
palliative (R1/R2)	45 (22,5)

(B) Summary of patient characteristics used for mRNA expression analysis of the target genes Gas6, Axl, Mer and Tyro3 in colorectal liver metastasis and normal liver tissue of each patient respectively (n = 24)	
	*n* (%) or median (IQR)
Total *n*	24 (100)
**Gender**	
male	15 (63)
female	9 (37)
**Age (years)**	64 (34–84)
**Tumor size**	
T1	1 (4,2)
T2	3 (12,5)
T3	15 (62,5)
T4	4 (16,7)
N.N.	1 (4,2)
**Lymph node status**	
positive	17 (70,8)
negative	6 (25)
N.N.	1 (4,2)
**Tumor differentiation**	
High (G1)	0 (0)
Moderate (G2)	15 (62,5)
Poor (G3)	6 (25)
N.N.	3 (12,5)
**Neoadjuvant therapy**	
Yes	5 (20,8)
No	16 (66,7)
N.N.	3 (12,5)
**Adjuvant therapy**	
Yes	17 (70,8)
No	3 (12,5)
N.N.	4 (16,7)
**Treatment**	
curative (R0)	14 (58,3)
palliative (R1/R2)	4 (16,7)
N.N.	6 (25)

Patients used for the expression analysis in colorectal liver metastasis and normal liver tissue are included in the analysis of colorectal cancer samples and normal mucosa as well.

Comparing patients' CRC liver metastases to normal liver tissue respectively (confront Table 1B for patient characteristics) we found that both Gas6 and Tyro3 were higher expressed within the metastases (*p* < 0,0001 and *p* < 0,0001) (Figure 1C, 1D), whereas Axl and Mer did not show a different expression pattern (Supplementary Figure S1C, S1D). The expression of Gas6 and Axl within the primary tumor and liver metastases was similar, whereas the expression of Tyro3 and Mer was higher within the primary tumor (Supplementary Figure S1E–S1H).

In summary Tyro3 is higher expressed in primary human colorectal tumors and liver metastases compared to normal tissues, whereas Gas6 is only higher expressed in liver metastases. Collectively, these data suggest a potential role for Gas6/TAM receptor (especially Tyro3) signaling in colorectal cancer and metastasis progression.

### Gas6 is expressed in human colorectal cancer cells and tumor-associated macrophages

**Figure 2:**
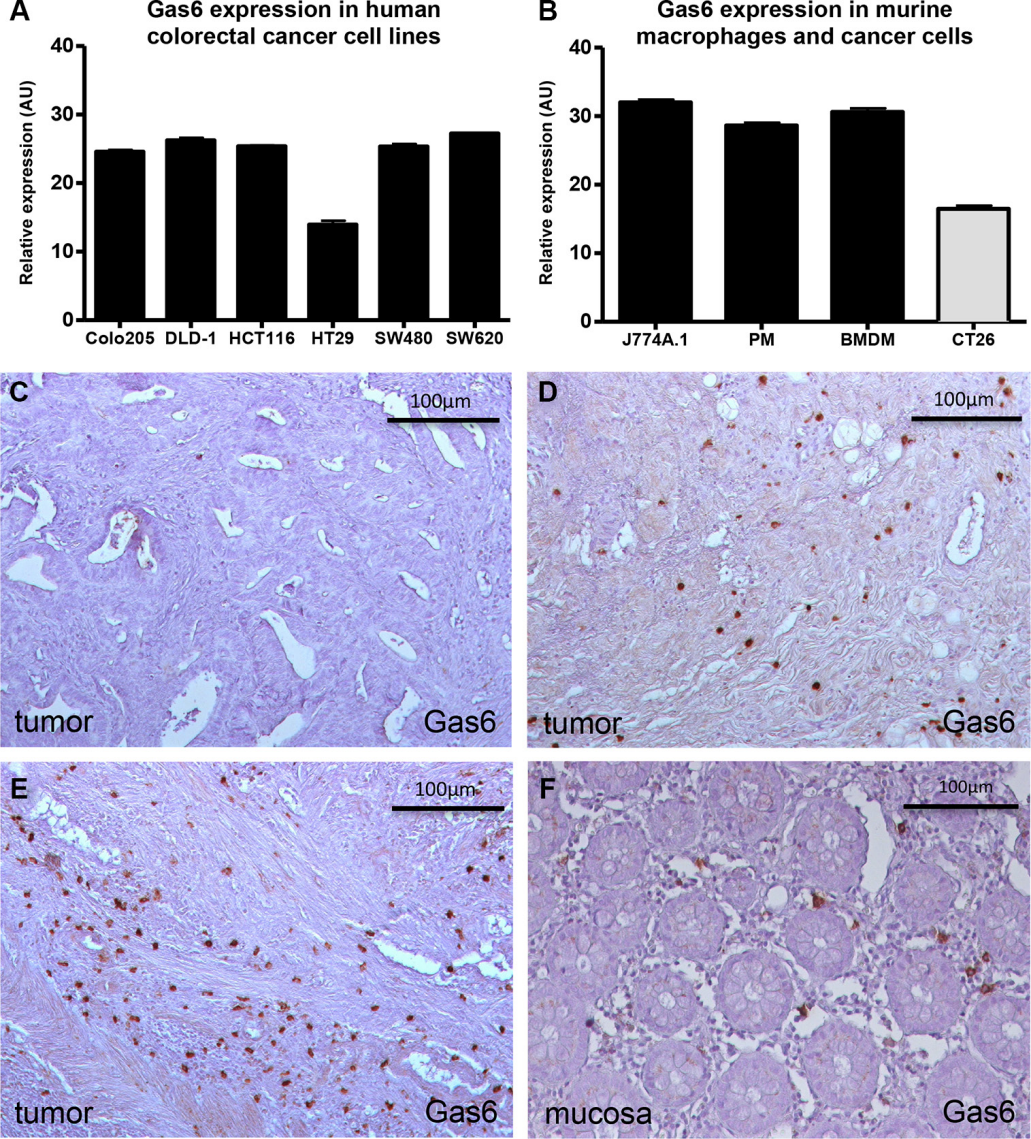
*In vitro* and *in vivo* expression of Gas6 (**A**) RT-PCR analyses showing similar Gas6 expression in different human colorectal cancer cell lines (*n* = 3; AU = 50 – ΔCp). (**B**) RT-PCR analysis showing higher Gas6 expression in murine macrophages (PM = peritoneal macrophages; BMDM = Bone marrow derived macrophages) compared to the murine colorectal cancer cell line CT26 (*n* = 3; AU = 50 – ΔCp). (**C**–**E**) Exemplary immunohistochemistry stainings for Gas6 in human colorectal cancer samples showing variable Gas6 expression (brown) (low to high = C–E) in tumor infiltrating cells (20 × magnification). (**F**) Exemplary staining for Gas6 in human colorectal mucosa sample showing Gas6 expression (brown) in infiltrating cells (20 × magnification). Scale bar = 100 μm.

As we observed expression of Gas6 and its receptors in human colorectal cancer samples, we proceeded to analyze the possible cellular origin. Thus, we first performed a qPCR analysis for Gas6 in HCT116, SW480, SW620, HT29, DLD-1 and Colo205 human colorectal cancer cell lines. Results indicate that all tested CRC cell lines expressed similarly low levels of Gas6 independent of their mutational status (i.e. KRAS, p53, BRAF and PIK3CA) (Figure 2A) (Supplementary Table S4). Murine CT26 tumor cell line displayed even lower expression of Gas6 (Figure 2B). In immunohistochemical stainings of human CRC samples, Gas6 was primarily expressed in tumor infiltrating immune cells (Figure 2C–2E). In addition, Gas6 was expressed by stromal cells in normal colon tissue of the same patients (Figure 2E). The number of Gas6 expressing cells within the tumor did not correlate with tumor stage or i.e. lymph node metastases (not shown).

**Figure 3:**
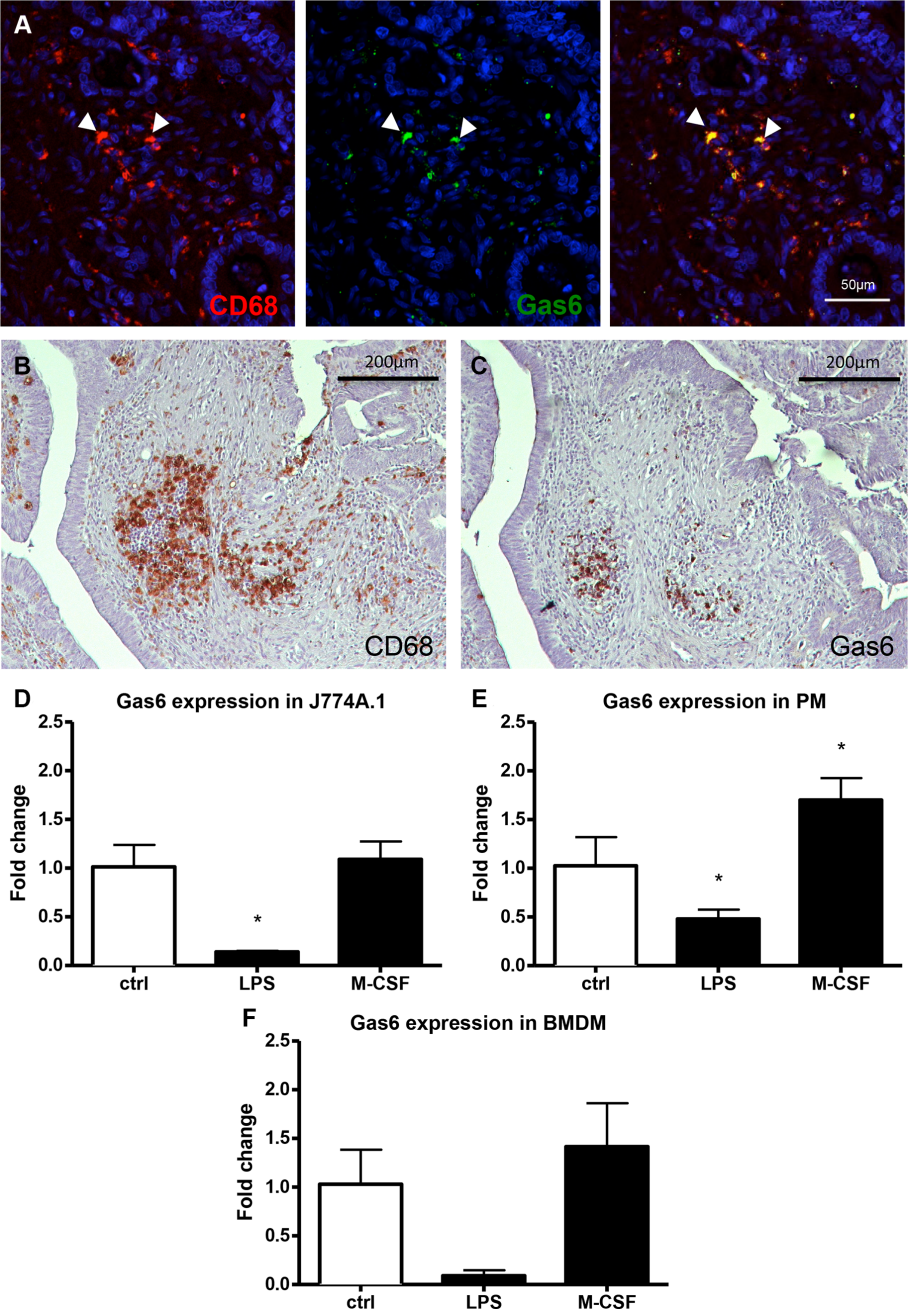
*In vivo* and *in vitro* expression of Gas6 in human and murine macrophages (**A**) CD68 (red) and Gas6 (green) double staining in human colorectal cancer tissue sample showing co-expression (orange) of the two antigens in tumor infiltrating macrophages (40 × magnification, scale bar = 50 μm). (**B**) Staining for CD68 as a macrophage marker in human colorectal cancer tissue sample showing infiltrating macrophages (brown; 10 × magnification, scale bar = 200 μm). (**C**) Sequential staining in the same tissue area for Gas6 in human colorectal cancer tissue sample showing Gas6 expression in tumor infiltrating cells. Gas6 co-localizes to CD68 macrophages in the previous section (brown; 10 × magnification, scale bar = 200 μm). (**D**–**F**) RT-PCR analysis showing a reduced Gas6 expression in murine macrophages differentiated to a M1 (LPS) phenotype and an increased Gas6 expression in a M2 (M-CSF) phenotype (*n* = 3, *P* < 0.01). (PM = peritoneal macrophages; BMDM = Bone marrow derived macrophages).

Gas6 expression in macrophages was confirmed by qPCR in a murine macrophage-like cell line, in mouse bone marrow derived macrophages (BMDM) and in mouse peritoneal macrophages (Figure 2B). To determine whether Gas6 is likewise expressed in macrophages within human colorectal cancer tissue, we performed stainings and double immunostainings for Gas6 and the macrophage marker CD68, revealing Gas6 expression in a subpopulation of macrophages (Figure 3A–3C and Supplementary Figure S2A). Thus we further determined the population of macrophages that express Gas6 by differentiation of the murine macrophage cell line (J774A.1) to a M1 or M2 phenotype by LPS or M-CFS treatment, respectively. Interestingly, we found that Gas6 expression was decreased in M1 macrophages, but not in M2 macrophages (*p* < 0.001) (Figure 3D–3F). M1 and M2 macrophage phenotypes were confirmed by differential regulation of phenotype-specific target genes (Supplementary Figure S2B, S2C).

Altogether, our results show that in human colorectal cancer Gas6 is expressed in tumor cells. Furthermore, in human and mouse colorectal cancer Gas6 is even higher expressed in a subset of tumor infiltrating macrophages. Our *in vitro* analyses indicate that Gas6 expression is decreased in M1 macrophages, while expression is preserved in M2 macrophages.

### TAM receptors are expressed in colorectal cancer cells

In order to further understand the possible role of Gas6 in human colorectal cancer, we next analyzed the expression of its receptors in tumor cells. All human colorectal tumor cell lines expressed the TAM receptors Axl, Tyro3 and Mer. The highest expression was observed for Axl, whereas Tyro3 and Mer were lower expressed (Supplementary Figure S3A–S3F). Same was confirmed in the murine cell line CT26 (Supplementary Figure S3G). Thus, colorectal tumor cells express both the ligand and its receptors.

### Gas6 supports colorectal tumor cell growth

**Figure 4:**
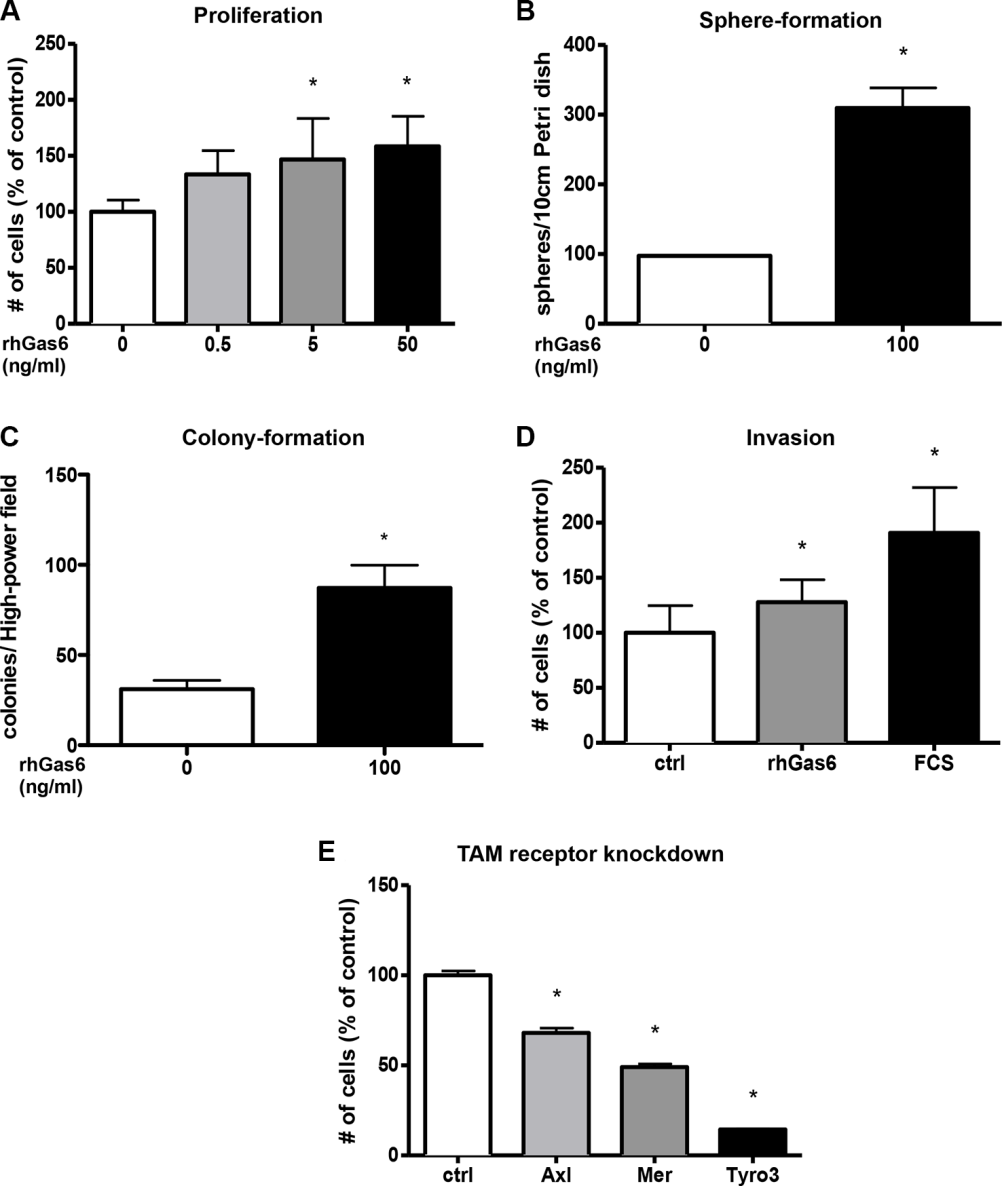
Gas6 induces proliferation, colony-, sphere-formation and invasion in human colorectal cancer cell lines *in vitro* (**A**) Human recombinant Gas6 (rhGas6) dose-dependently induces proliferation of human colorectal cancer cells (HCT116) (*n* = 6; *P* < 0.05). (**B**) Colorectal cancer cells (HCT116) treated with 100 ng/ml recombinant human Gas6 form significantly more spheres after 10 days compared to control treated cells (*n* = 3; *P* < 0.05). (**C**) Colorectal cancer cells (HCT116) treated with 100 ng/ml recombinant human Gas6 form significantly more colonies after 10 days compared to control treated cells (*n* = 3; *P* < 0.05). (**D**) Invasion of human colorectal cancer cells (HCT116) is increased after 24 hours treatment with recombinant human Gas6 (rhGas6) *in vitro* (*n* = 9; *P* < 0.05). 20% FCS was used as positive control (*n* = 9; *P* < 0.05). (**E**) siRNA knockdown of the TAM receptors in human colorectal cancer cells (HCT116) lead to an significantly decreased proliferation. The most distinct effect was observed for Tyro3 > Mer > Axl (*n* = 6; *P* < 0.05).

As Gas6 has been shown to induce proliferation in different cell types we assessed if Gas6 is also mitogenic for human colorectal cancer cells. Indeed, recombinant Gas6 dose-dependently induced proliferation in HCT116 colorectal tumor cells *in vitro* (*p* < 0.005) (Figure 4A). Similarly proliferation was induced in other CRC cell lines such as SW480 and SW620 (*p* < 0.01 and *p* < 0.01) (Supplementary Figure S4A, S4B). Moreover, Gas6 stimulation induced colony and sphere formation of CRC cells (*p* < 0.001 and *p* < 0.01) (Figure 4B, 4C and Supplementary Figure S4C–S4F). Using a transwell assay we found that Gas6 did not increase migration of tumor cells (Supplementary Figure S4G), however invasion was significantly increased by Gas6 stimulation (*p* < 0.05) (Figure 4D).

Altogether, these results indicate that Gas6 is able to induce proliferation, colony formation and invasion of CRC tumor cells and suggest that within CRC tumors, Gas6 secreted either by tumor cells or macrophages, might regulate these processes in an autocrine or paracrine manner, respectively.

To further elucidate the significance of the different TAM receptors in CRC cell lines, we specifically knocked down Axl, Mer and Tyro3 via siRNA transfection. These experiments showed that Mer and Tyro3 knockdown strongly suppressed the proliferation of CRC cells by 51% and 86% respectively (*p* < 0.0001 and *p* < 0.0001), both to a higher extent than Axl knockdown (*p* < 0.0001)(Figure 4E). These data indicate that Mer and Tyro3 seem to play a previously unknown important role in CRC.

### Mer and Tyro3 expression influence the survival of CRC patients

To confirm the importance of our *in vitro* findings we correlated the expression of Gas6 and the TAM receptors in human samples with the patients' outcome. In this way we aimed to elucidate if Gas6 or the TAM receptors might present a novel therapeutic target in CRC. Gas6 or its receptors were not differentially expressed according to UICC tumor stages. However, Axl expression within the tumor in comparison to normal colonic mucosa was higher with increasing T category (T4 > T3 > T2 > T1) (*p* = 0.028). Similarly, Gas6 was higher expressed in patients with lymph node metastases, while Mer expression showed a trend towards higher expression in primary tumors from patients suffering liver metastasis (*p* = 0.1).

**Figure 5:**
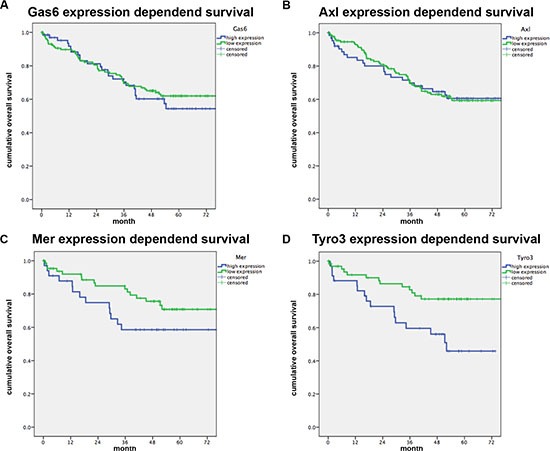
Gas6 and TAM receptor expression correlated to patients survival RT-PCR analysis was performed for Gas6, Axl, Mer and Tyro3 in colorectal tumor samples and normal mucosa. Kaplan-Maier survival analyses were performed for patients with high gene expression (blue) (highest 33%) and low gene expression (green) in the tumor vs normal mucosa (ΔΔC *p* values).(**A, B**) Relative Gas6 and Axl mRNA expression is not associated with patients survival (*n* = 200; *P* = NS). (**C**, **D**) Patients with a high Mer or Tyro3 mRNA expression in the tumor have a significantly shorter survival (*n* = 103; *P* < .05).

When analyzing patients' overall survival it was found that neither Gas6 nor Axl expression in primary human colorectal tumors influenced overall survival or recurrence free survival (Figure 5A, 5B and Supplementary Figure S5A, S5B). By contrast, expression of both Tyro3 or Mer were associated with reduced overall (Figure 5C, 5D; *p* < 0.01 and *p* < 0.05) and metastases free survival (Supplementary Figure S5C, S5D), once more underlying the apparent important role of these receptors in CRC.

### Gas6 expression is increased in macrophages upon chemotherapy and partially blocks the chemotherapeutic effect

**Figure 6:**
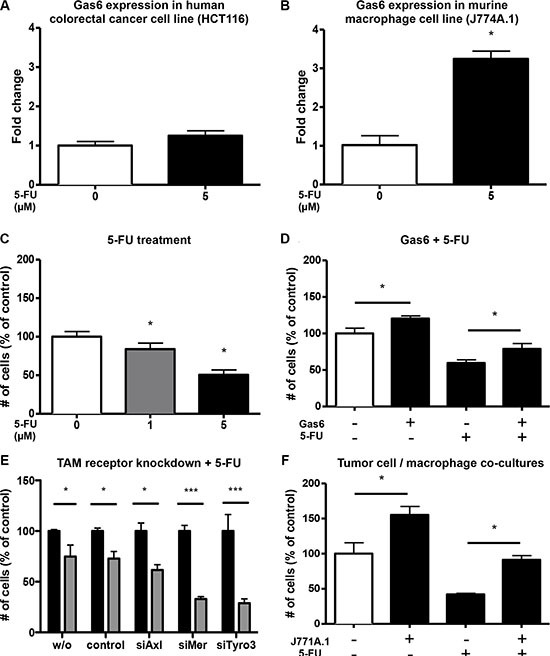
Macrophages upregulate Gas6 under 5-FU treatment and mediate a chemoprotective effect (**A**) RT-PCR analysis revealing no upregulation of Gas6 in human colorectal cancer cells (HCT116) after 24 hours 5-FU treatment (*n* = 3; *P* = NS). (**B**) RT-PCR analysis revealing significant upregulation of Gas6 in murine macrophages (J774A.1) after 24 hours 5-FU treatment (*n* = 3; *P* < .05). (**C**) 5-Fluorouracil dose-dependently reduces proliferation of human colorectal cancer cells (HCT116) *in vitro* after 48 hours treatment (*n* = 6; *P* < .05). (**D**) The antiproliferative effect of 5-Fluorouracil was partially antagonized by Gas6 in colorectal cancer cells (HCT116) treated with recombinant human Gas6 (100 ng/ml) and 5-Fluorouracil (5 μM) *in vitro* (*n* = 6; *P* < .05). (**F**) Co-cultures of murine colorectal cancer cells (CT26) with murine macrophages (J774A.1) reveal a chemoprotective effect of the macrophages likely mediated by Gas6 (*n* = 3, *P* < .05).

As many patients with advanced colorectal cancer are treated with a 5-FU containing chemotherapy regimen, we assessed the influence of 5-FU on the expression of Gas6 and its receptors in CRC tumor cells and macrophages. 5-FU treatment of the CRC tumor cell line HCT116 did not lead to any changes in Gas6 expression (Figure 6A), while Gas6 expression was increased in the macrophage cell line J774A.1 upon 5-FU treatment (*p* < 0.0005) (Figure 6B).

Proliferation of HCT116 cells was reduced upon 5-FU treatment (*p* < 0.0001) (Figure 6C). However, when Gas6 protein (100 ng/ml) was added as a co-treatment, HCT116 proliferation was increased compared to the proliferation of cells treated with 5-FU only (Figure 6D). When knocking down Tyro3 or Mer, the anti-proliferative effect of 5-FU was enhanced leading to an inhibition by 67.1% and 71.2 % instead of 27.2% in control siRNA transfected cells, and of 38.4% in cells transfected with siRNA for Axl (*p* < 0.05) (Figure 6E).

We next sought to determine whether in a tumor cell / macrophage co-culture, 5-FU treatment would cause similar effects as outlined above. Thus, we co-cultured CT26 colon tumor cells and J774A.1 macrophages in the presence of 5-FU, and assessed the proliferation of CT26 cells. As shown before, tumor/macrophage co-culture resulted in increased proliferation of tumor cells (Figure 6F). 5-FU inhibited the proliferation of CT26 cells, however, the presence of macrophages partially blocked the inhibition mediated by 5-FU treatment through increased Gas6 expression (Figure 6F).

Altogether, these data suggest that macrophage-derived Gas6 confers resistance to 5-FU containing chemotherapy.

### Protein S induces proliferation but does not influence patients' survival

**Figure 7:**
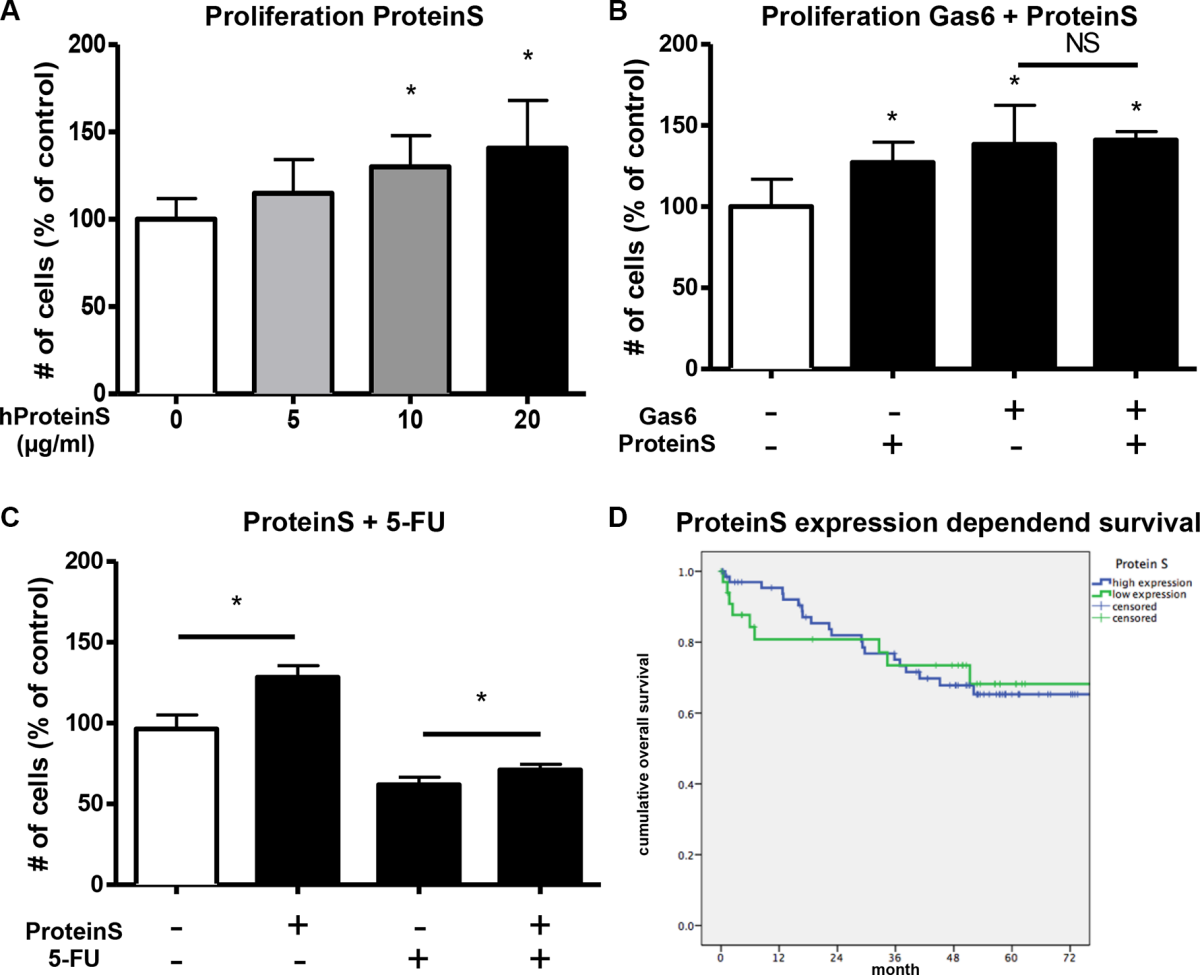
ProteinS increases proliferation and chemoresistance *in vitro* (**A**) Human ProteinS (hProteinS) dose-dependently induces proliferation of human colorectal cancer cells (HCT116) *in vitro* after 48 hours treatment (*n* = 6; *P* < .05). (**B**) Simultaneous stimulation of human colorectal cancer cells (HCT116) with recombinant human Gas6 (100 ng/ml) and human ProteinS (20 μg/ml) *in vitro* with no additive proliferative effect of both mitogens together (*n* = 6; *P* < .05). (**C**) Simultaneous treatment of human colorectal cancer cells (HCT116) with human ProteinS (20 μg/ml) and 5-Fluorouracil (5 μM) *in vitro*. The antiproliferative effect of 5-Fluorouracil was partially antagonized by Protein S (*n* = 6; *P* < .05). (**D**) RT-PCR analysis was performed for ProteinS in colorectal tumor samples and normal mucosa of the patients. No difference in Kaplan-Maier analyses of overall survival in patients with high (blue) or low (grean) expression within the tumor. Relative ProteinS mRNA expression is not associated with patients survival (*n* = 103; *P* = NS).

As Protein S is known to signal through the Tyro3 and Mer receptors, we additionally checked whether Protein S could play a role in CRC. Indeed, we found that Protein S dose-dependently induced proliferation of CRC cells *in vitro* (Figure 7A and Supplementary Figure S6A, S6B). When adding both Gas6 and Protein S no additional proliferative effect was observed (Figure 7B). Protein S was similarly to Gas6 able to reduce the efficacy of 5-FU treatment *in vitro* (Figure 7C). However, also for Protein S no influence on the overall survival of patients was found (Figure 7D and Supplementary Figure S6C).

## DISCUSSION

This study provides evidence for the role of the Gas6/TAM receptor pathway, especially the Mer and Tyro3 receptors in human colon cancer. The key findings of the study are: (1) Gas6, Protein S and the TAM receptors are expressed in colon cancer with a high expression of Tyro3 in the primary tumors and liver metastases and a high Gas6 expression within the metastases; (2) Gas6 is derived from tumor infiltrating M2 macrophages and induces tumorigenic processes (proliferation, invasion and sphere and colony-formation) in colon cancer cells; (3) Gas6 and Protein S both confer resistance to chemotherapy, while not showing additive effects to each other; (4) the TAM receptors Tyro3 and Mer play a before unknown important role in CRC with their significance for tumor cell proliferation and chemotherapy resistance; and finally (5) high expression of Mer and Tyro3 significantly correlate to a reduced overall survival of CRC patients.

While the role of Gas6 and the TAM receptors has already been studied in different diseases, the current role in CRC was so far not clearly defined. In a preclinical CRC tumor model it was previously shown that Gas6 is expressed in macrophages [20]. Similarly in our study Gas6 expression in human tumor samples was mainly found in a subpopulation of CD68^+^ macrophages. Analogous this expression of Gas6 in macrophages was also observed in human samples of non small cell lung cancer [19]. However until now it was not clearly defined if expression of Gas6 is dependent on M1 or M2 macrophages. In colorectal cancer both phenotypes are usually present within the same tumor sample with a higher amount of M2 differentiated macrophages at the invasive front [30]. In this study we show that murine M2 macrophages express significantly higher levels of Gas6 compared to M1 macrophages. In contrast to the preclinical findings [20] in human CRC Gas6 is also expressed to a low level in several different CRC tumor cells *in vitro*, independent of their origin and mutational status [20]. Similarly Martinelli et al. showed mRNA expression of Gas6 in CRC cell lines while not observing protein levels in culture medium, suggesting that Gas6 is not secreted by CRC cells [31]. However, their conclusion that the Axl receptor is ligand-independently activated [31] should be reconsidered. As shown in this study, Gas6 is strongly expressed in macrophages and a macrophage-tumor communication via Gas6 could be acting on Axl. Although Gas6 expression is increased in murine M2 macrophages it needs to be noted that severe differences between human and murine macrophages exist [32–34], which makes a direct comparison between them difficult. Thus our conclusion that Gas6/CD68 positive cells in our *in vivo* double stainings are also M2 macrophages is not ultimately confirmed and remains a limitation.

Our results of the functional role of Gas6 in human CRC progression are similar to previous findings in murine models [20]. Additionally, we were able to show that Protein S is expressed in human CRC samples and induces proliferation to a similar extend as Gas6. Protein S expression was shown in other malignancies like malignant lymphoma [35], NSCLC [36] and SCLC [37] while only weak expression is shown in others [29]. Similar to our findings, Protein S has been shown to induce migration in prostate cancer cells [38]. Our data suggest an additional role of Protein S in tumor progression, beside its well known function in the coagulation process, that should be studied in more detail in the future. However, even though we found that Gas6 and Protein S are protumorigenic *in vitro*, their expression did not show any difference in overall patient survival *in vivo*. This underlines our conclusion that the expression of different TAM receptors might be more important than the presence of Gas6 in human CRC.

The expression of Axl was reported in CRC patients and Axl was suggested as a novel oncotarget [31]. It was shown that foretinib can dose dependently inhibit CRC cells and tumor growth in mice and that siRNA knockdown for Axl reduces viability of CRC cells by approximately 22–32%, which is exactly in line with our findings [31]. It might have been overlooked that the highest efficacy of foretinib is against the Mer receptor, potentially leading to a different interpretation considering our novel data. Even tough others described promising antitumor effects in preclinical models and an overexpression of Axl was found in CRC, we found no correlation with CRC patients' survival [31]. In one study subgroup analyses revealed that high Axl expression has a negative prognostic effect in early stage II CRC patients, but not in stage III or a combination of stage II/III patients [39]. This supports our findings, in which Axl expression neither affects overall nor recurrence free survival of CRC patients including all UICC stages (I–IV), questioning whether Axl should be considered as a therapeutic target in CRC. It is also important to mention that the Axl and Mer receptor have an anti-inflammatory function with a paradox effect in colorectal cancer. In a chemically induced inflammation associated colorectal cancer model the tumors exacerbated in mice lacking Axl and Mer [40]. Therefore especially in colitis associated colorectal cancer the inhibition of Axl should only be considered with caution.

Finally we found that the rather overlooked receptors Tyro3 and Mer play an important role in CRC. Even tough being expressed at lower level than the Axl receptor, knockdown of the receptors, especially of Tyro3 leads to a significant inhibition of proliferation *in vitro*. While Gas6 can attenuate the effect of 5-FU therapy *in vitro*, knockdown of Tyro3 and Mer even enhances its efficacy. For Mer a transforming effect is known in the hematopoietic system, where overexpression leads to the development of lymphadenopathy and T-lymphoblastic leukemia [41]. Mer was suggested as a novel target in multiple myeloma, acute lymphoblastic leukemia, per-B-cell acute lymphblastic leukemia, acute myeloid leukemia and lately also in glioblastoma multiforme [21–24, 42]. Similar to our data it was shown that Mer inhibition may reduce survival and chemotherapy resistance in tumor cells. Initial data was generated in hematological and diseases of the central nervous system and recently complemented by findings in gastric cancer [23, 43, 44]. Our data is in line with these findings and for the first time suggests Mer as a target in CRC.

Tyro3, with its low expression in CRC, was so far not studied at all in CRC. In ovarian cancer it is known that Tyro3 overexpression analogously can lead to resistance against taxol based therapy [45] and that its inhibition can circumvent this resistance [46, 47]. Apart from ovarian cancer Tyro3 was suggested as target in breast cancer, melanoma and thyroid cancer [26–28, 48]. All these findings have in common that they are mainly based on *in vitro* findings. Beside our *in vitro* findings, that showed a significant inhibition of tumor cell proliferation and increase sensitivity to 5-FU chemotherapy after Tyro3 knockdown in human CRC cells, we where able to show a relevant role *in vivo*. As our data show, Tyro3 is overexpressed in patients with CRC as well as in liver metastases in comparison to normal liver. This opens the question if Tyro3 expression is actually essential for CRC and how its expression is regulated, as it was not dependent on the mutational status of the different cell lines *in vitro*. Additionally we were able to show a negative association between the Tyro3 expression and patients' survival, underlining a so far unknown role of Tyro3 as a potential new oncotarget.

In summary our study identified the Gas6/TAM receptor pathway with Tyro3 and Mer as novel targets in CRC. While specific Axl inhibitors are already in clinical phase I trials [49], Mer is only inhibited besides other molecules by amuvatinib [50] and no clinical trial is known to us targeting Tyro3 [51]. Our study warrants further research into Tyro3 and Mer as targets in CRC.

## MATERIALS AND METHODS

### RT-qPCR

All human tissue samples were stored at −80°C. mRNA isolation was performed with the RNeasy Mini Kit (Qiagen) following the company's protocol with a supplementary DNA digestion using the RNase-Free DNase Set (Qiagen). Isolated mRNA concentration and quality was measured with the NanoDrop 2000 (Peqlab). ImProm II Reverse Transcriptase System (Promega) was used for the following cDNA synthesis made of 500 ng mRNA respectively. Reverse transcription was performed in the Mastercycler gradient (Eppendorf) with random primers according to the company's protocol. cDNA was diluted 1:10 in nuclease-free water and stored at −20°C. Finally quantitative reverse-transcription polymerase chain reaction (RT-PCR) was performed by use of primers listed in Supplementary Table S1 for all patients listed in Supplementary Table S2 [52]. Relative expression was assessed by calculation of ΔCp and ΔΔCp. Fold change calculation for *in vitro* assays was performed as described previously [53].

### Immunostaining

Human paraffin-embedded tissue samples listed in Supplementary Table S3 were cut into 5 μm sections and stained for CD68 (monoclonal mouse anti-human CD68; Dako) and Gas6 (polyclonal goat anti-human Gas6; Santa Cruz Biotechnology) respectively. Primary antibodies were diluted 1:100 and incubated over night at 4°C. Biotinylated secondary antibodies (biotinylated horse anti goat antibody or biotinylated horse anti mouse antibody; Vector Laboratories) were used along with the TSA^™^ Biotin System Kit (Perkin-Elmer) for signal amplification according to the company's protocol. Secondary antibodies were diluted 1:400 and incubated for 30 min. Visualization of the antigen-antibody complex was performed with DAB-Chromogen (Dako). Hematoxylin was used for a 3 sec counterstaining and Eukitt (Kindler) as permanent mounting medium. For all analyses, at least 10 optical fields (20 × magnification) per tumor were randomly chosen and analyzed regarding the number of positive stained cells.

For immunofluorescence, human tissue samples were rehydratated and treated with a trypsin solution (5 mg trypsin; Sigma-Aldrich, in 1% CaCl2 solution in destilated water) for antigen retrieval. Samples were blocked for 1 hour at RT (1% BSA, 0.3% TritonX100 in PBS) and incubated with the primary antibodies over night at 4°C in blocking solution. Fluorescent-conjugated secondary antibodies (donkey anti-goat A488 and goat anti-mouse A568; Life Technologies) were incubated for 2 hours at RT and Fluoromont-G (Southern Biotech) was used for mounting. Samples were imaged on a confocal microscopy (Zeiss LSM 510 META) using a 1.1NA ×40 oil-immersion objective.

### *In vitro* assays

### RT-PCR, mRNA analysis

Baseline mRNA expression analysis was assessed for cell lines listed in Supplementary Table S4 by use of primers listed in Supplementary Table S5 following the previously described RT-PCR protocol. Cells were cultured in specific medium supplemented with 10% FCS for 24 h in 24 well plates. 100.000 cells / well were seeded in triplicates respectively. Additionally cells were treated with 5-FU (0–10 μM) for 24–48 hours and analyzed for the Gas6 mRNA Expression.

### Isolation and differentiation of peritoneal and bone marrow derived macrophages

Peritoneal macrophages were isolated by flushing the peritoneal cavity of 10-week-old mice with cold PBS buffer (Dulbecco's PBS without Mg^+^ & Ca^2+^; PAA) and allowed them to become adherent overnight in RPMI medium (RPMI 1640; PAA) supplemented with 10% FCS. Subsequently cells were differentiated in M1 or M2 phenotype by a 4 hours treatment with 1 μg/ml LPS (Sigma-Aldrich) or 10 ng/ml M-CSF (Sigma-Aldrich) respectively [54].

Bone marrow was isolated by rinsing the shaft of femur and tibia of the same mice with PBS buffer (Dulbecco's PBS without Mg^+^ & Ca^2+^, PAA) and a 27G needle. Hematopoietic stem cell were cultured in DMEM medium (DMEM High glucose; PAA) supplemented with 10% FCS and differentiated in Macrophages with a five days treatment of 50 ng/ml M-CSF (Sigma-Aldrich). Afterwards cells were differentiated in M1 or M2 phenotype by a 48 hours treatment with 1 μg/ml LPS (Sigma-Aldrich) or 10 ng/ml M-CSF (Sigma-Aldrich) respectively [54].

Same was performed with the macrophage cell line J774A.1 following the previously described protocol. M1 and M2 macrophage phenotype was confirmed by RT-PCR expression analysis of M1 (iNOS and IL-6) and M2 (Arginase and CCR2) specific genes using the previously described protocol and the primers listed in Supplementary Table S6 [55].

### *In vitro* proliferation assay

Human colorectal cancer cells (HCT116, SW480 and SW620) harvested from subconfluent cultures were seeded at 5.000 cells/well in 96-well microplates in medium with 1% FCS and incubated with different concentrations and combinations of rhGas6 (0–100 ng/ml; R&D Systems), hProteinS (0–20 μg/ml; Enzyme Research Laboratories) and 5-FU (0–5 μM). After 48 hours treatment, proliferation was measured using the Cell Proliferation Reagent WST-1 (Roche) and normalized to the signal obtained when using a standard row of cells, seeded at defined densities according to the company's manual.

### *In vitro* colony- and sphere-forming assay

Human colorectal cancer cells (HCT116) harvested from subconfluent cultures were seeded at 5.000 cells/10 cm culture dish in 10ml serum-free medium with or without rhGas6 (100 ng/ml; R&D Systems). Cells were cultured for 10 days and subsequently stained for 10 min with 0.05% Crystal violet solution diluted in 20% ETOH. Colonies were count macroscopically and spheres were count microscopically (10 × Magnification). Exemplary pictures were taken from each group (Supplementary Figure S4C–S4F).

### *In vitro* migration and invasion assay

Migration and Invasion assays were performed using a transwell method (ThinCert – 24 well; pore size 8 μm; greiner-bio one), with Matrigel^™^ Basement Membrane Matrix (Becton Dickinson) coated membranes for invasion assays. 10.000 colorectal cancer cells (HCT116)/well were seeded in serum-free medium and migration/invasion was enabled for 24 hours. Recombinant human Gas6 (100 ng/ml; R&D Systems) and 20% FCS supplemented medium worked as chemoattractants whereas FCS was used as a positive control. Migrated cells were stained with a Crystal violet solution. Subsequently staining was extracted from the cells by 10% acetic acid and absorption of the extracted solution was measured using an ELISA-Reader at 540 nm (TECAN).

### TAM receptor knockdown

The TAM receptors Axl, Mer and Tyro3 were knocked down in human colorectal cancer cells (HCT116) by previously confirmed siRNAs listed in Supplementary Table S7 using Lipofectamin RNAi MAX (invitrogen). Cell transfection was performed for 24 hours in 12-well plates in antibiotics-free DMEM Ham's F-12 medium. Transfected cells were seeded at 10.000 cells/well in 96-well microplates and proliferation was assessed after 48 hours using the Cell Proliferation Reagent WST-1 (Roche).

### Cancer cell/macrophage co-culture

Murine colorectal cancer cells (CT26) and macrophages (J774A.1) were cocultured in indirect contact using cell culture inserts (pore size 1 μm; Becton Dickinson) in DMEM high glucose medium (supplemented with 1% FCS; PAA). 48 hours later, proliferation was assessed in the upper compartment (CT26) with the Cell Proliferation Reagent WST-1 (Roche). Gas6 mRNA expression of the macrophages was assessed by RT-PCR following the previously described protocol.

### Statistics

*In vitro* data represent mean ± SEM of representative experiments unless otherwise stated. Statistical significance was calculated by a Student *t* test except for tumor growth kinetics, where analysis of variance was used (Prism Version 5.0 b; GraphPad Software).

We measured overall survival from point of surgery until death or loss of follow up. The Kaplan Meier Method was performed for survival analysis, for differences in survival time we used the log-rank test. Patient data and material was collected in a prospective database. Results for overall survival were confirmed with multi-variate Cox regression analysis. Statistical significance was taken as a *p*-value of < 0.05 (two-tailed). All statistical analyses were done using SPSS software version 20.0 (SPSS, Inc., Chicago, Illinois, USA).

## SUPPLEMENTARY MATERIALS FIGURES AND TABLES


