# Supplementary material for: TAM receptors Tyro3 and Mer as novel targets in colorectal cancer
Source: Oncotarget. 2016 Jul 28;7(35):56355–70. doi: 10.18632/oncotarget.10889 (PMC5302919; doi:10.18632/oncotarget.10889)
Supplement: Supplementary file 1 [file oncotarget-07-56355-s001.pdf]

# TAM receptors Tyro3 and Mer as novel targets in colorectal cancer

## Supplementary Materials

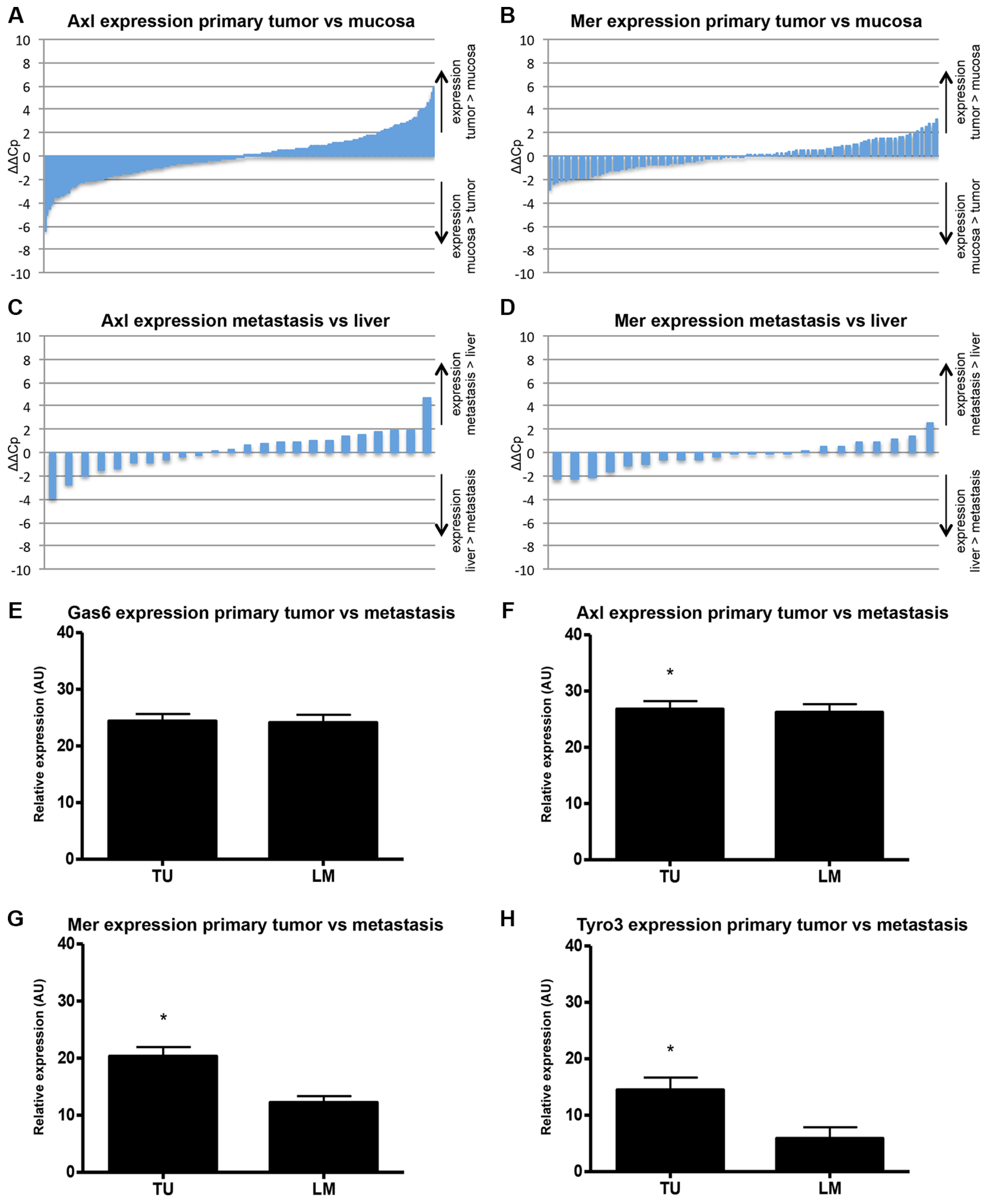


**Supplementary Figure S1: *In vivo* mRNA expression analysis of human tissue samples.** (**A–D**) Each bar represents one patient. Positive bars represent higher expression in tumor or metastasis compared to normal mucosa or normal liver tissue. Negative bars represent higher expression in mucosa or normal liver tissue compared to tumor or liver metastasis. (A) Axl mRNA expression in human CRC tumor samples and normal mucosa of the same patients respectively ($n = 200$; $P$ = NS). (B) Mer mRNA expression in human CRC tumor samples and normal mucosa of the same patients respectively ($n = 103$; $P$ = NS). (C) Axl mRNA expression in human CRC liver metastases and normal liver tissue of the same patients respectively ($n = 24$; $P$ = NS). (D) Mer mRNA expression in human CRC liver metastases and normal liver tissue of the same patients respectively ($n = 24$; $P$ = NS). (**E–H**) Average Gas6 ($P$ = NS), Axl ($P < .05$), Mer ($P < .05$) and Tyro3 ($P < .05$) mRNA expression in tumor (TU; $n = 200$) and liver metastasis (LM; $n = 24$; AU = 50 – ΔCp).

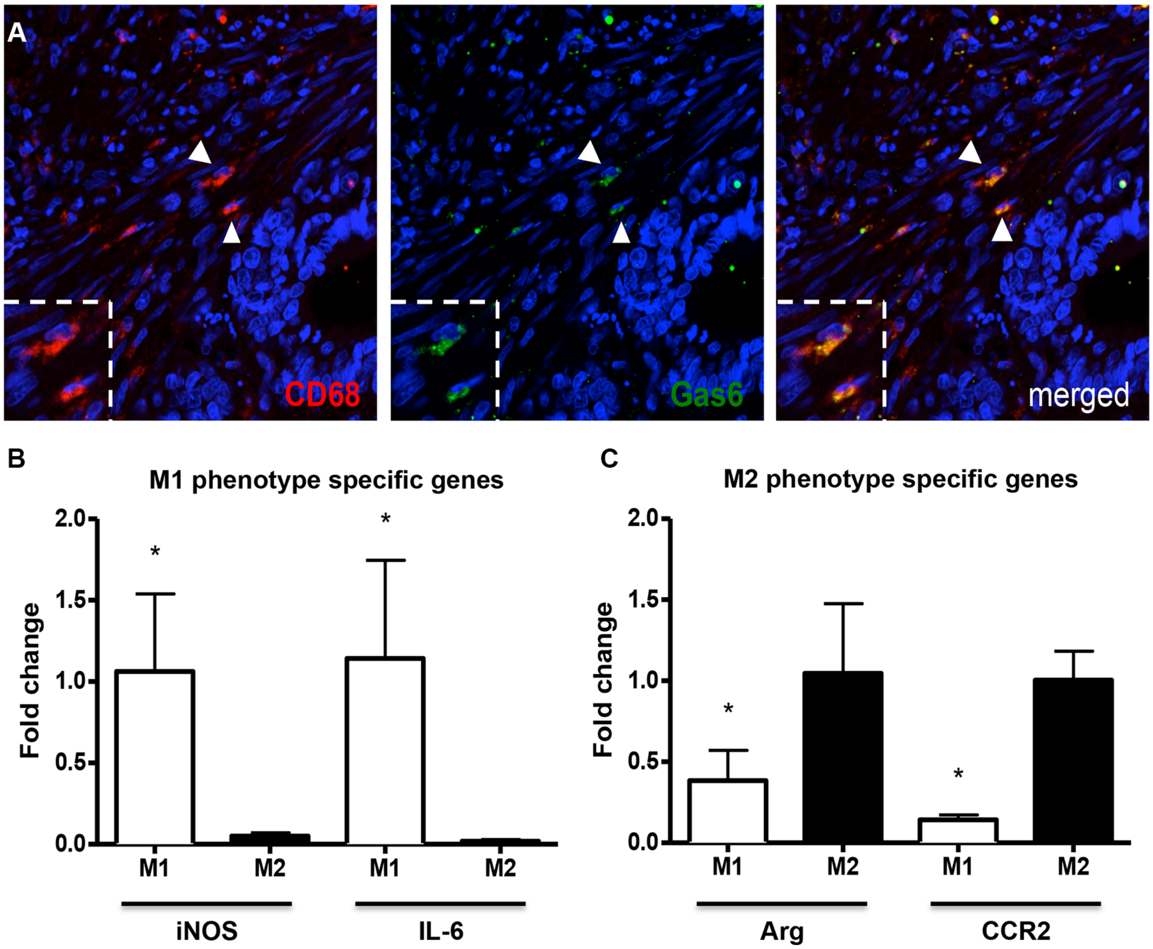


**Supplementary Figure S2: Gas6/CD68 double immune staining and RT-PCR analysis for verification of macrophage M1 and M2 phenotype.** (**A**) Exemplary immunofluorescence stainings for Gas6 (red) and CD68 (green) in human colorectal cancer samples showing co-localized expression in tumor infiltrating macrophages (orange). White arrows indicate double stained cells. (**B**, **C**) Macrophage cell line J774A.1 was used for verification of the differentiation towards an M1 or M2 phenotype by LPS (1 µg/ml) or M-CSF (10 ng/ml) treatment respectively. (A) mRNA expression of iNOS and IL-6 as two M1 phenotype specific genes is significantly higher in LPS (M1) compared to M-CSF (M2) treated macrophages ($n = 3$; $P < .05$). (B) mRNA expression of Arginase (Arg) and CCR2 as two M2 phenotype specific genes is significantly higher in M-CSF (M2) compared to LPS (M1) treated macrophages ($n = 3$; $P < .05$).

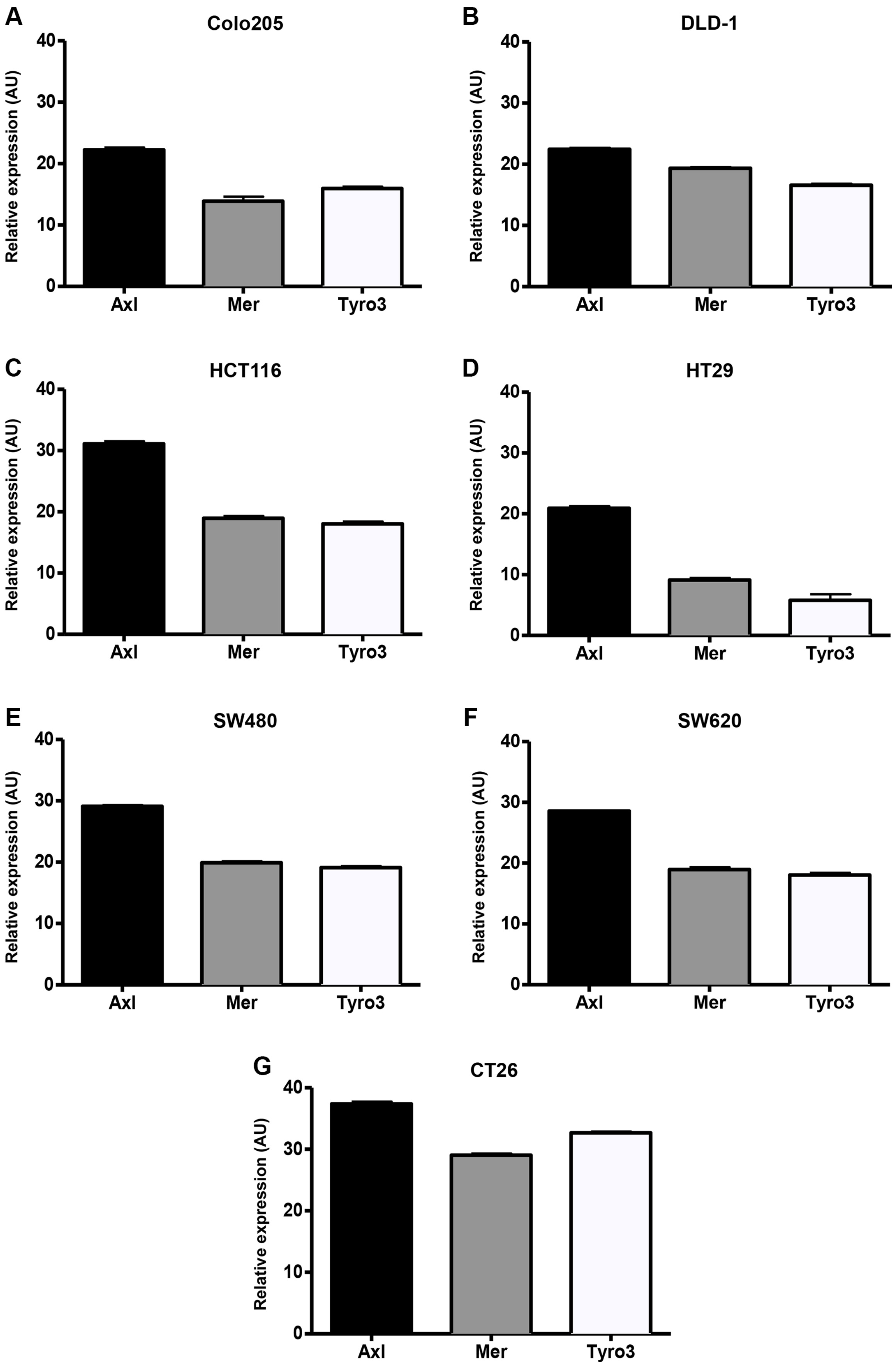


**Supplementary Figure S3: TAM receptor expression in human and murine colorectal cancer cell lines.** RT-PCR technique was used to analyze the TAM receptor expression levels of different human and murine colorectal cancer cell lines. (**A**–**F**) Axl, Mer and Tyro3 mRNA expression in human colorectal cancer cell lines ($n$ = 3; AU = 50 – ΔCp). (**G**) Axl, Mer and Tyro3 mRNA expression in the murine colorectal cancer cell line CT26 ($n$ = 3; AU = 50 – ΔCp).

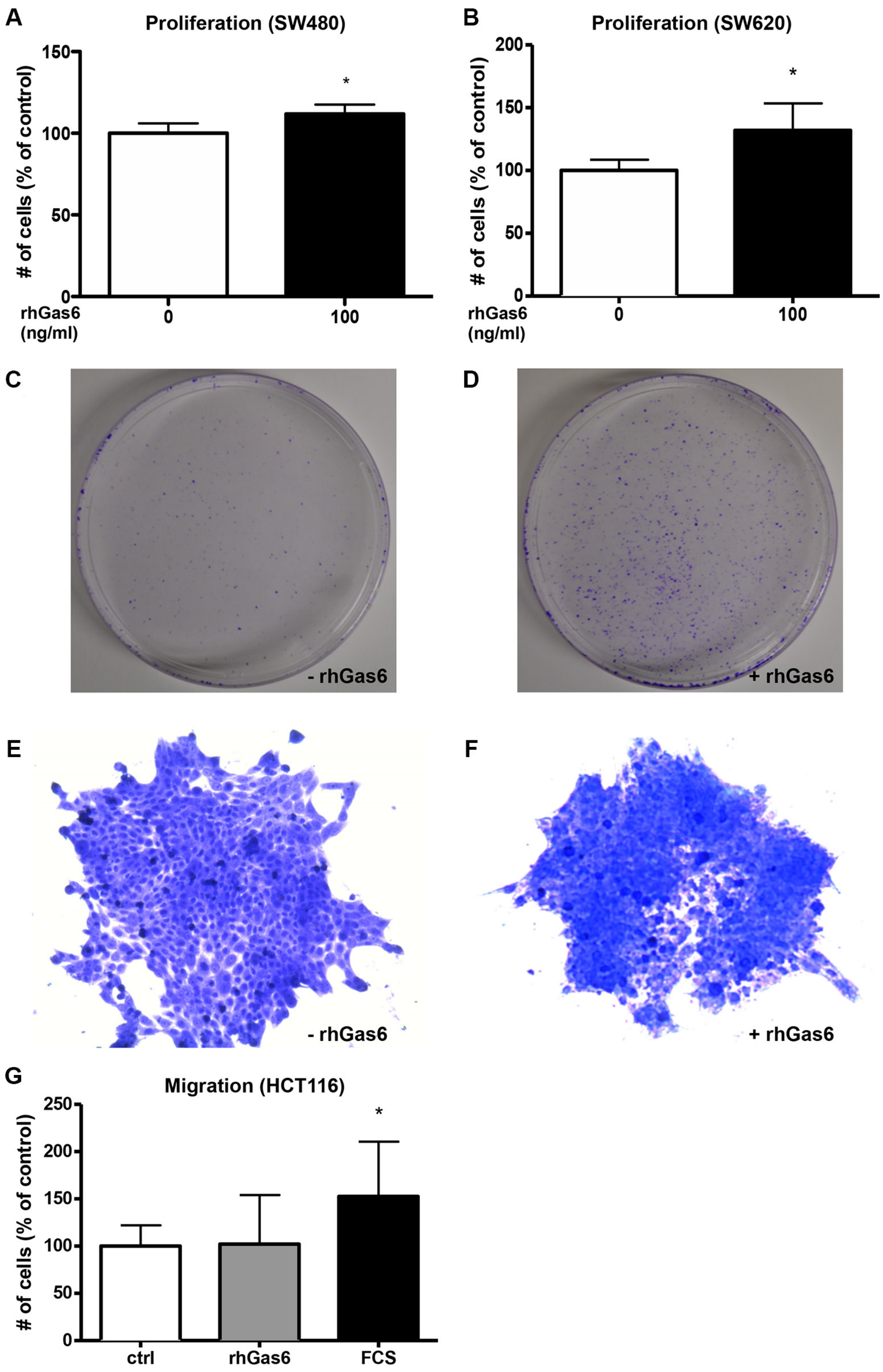


**Supplementary Figure S4: Gas6 induces proliferation, colony- and sphere-formation in human colorectal cancer cell lines *in vitro*.** (**A**, **B**). Human recombinant Gas6 (rhGas6) induces proliferation of human colorectal cancer cell lines (SW480 and SW620) *in vitro* after 48 hours treatment ($n$ = 6; $P$ < .05). (**C**) Colony-forming assay; control treated colorectal cancer cells (HCT116). Only few colonies were formed after 10 days. (**D**) Colony-forming assay; colorectal cancer cells (HCT116) treated with 100 ng/ml recombinant human Gas6. Significantly more colonies were formed after 10 days compared to control treated cells. (**E**) Sphere-formation assay; control treated colorectal cancer cells (HCT116). Cancer cells were grown mainly in monolayer and were less likely to build spheres after 10 days. (**F**) Sphere-formation assay; colorectal cancer cells (HCT116) treated with 100 ng/ml recombinant human Gas6. Significantly more spheres were formed after 10 days compared to control treated cells. (**G**) Migration assay using a transwell method showing no influence of Gas6 concerning tumor cell migration after 24 hours treatment of human colorectal cancer cells (HCT116) with 100 ng/ml recombinant human Gas6 (rhGas6) *in vitro* ($n$ = 12; $P$ = NS). 20% FCS was used as a positive control showing significantly increased migration ($n$ = 12; $P$ < .05).

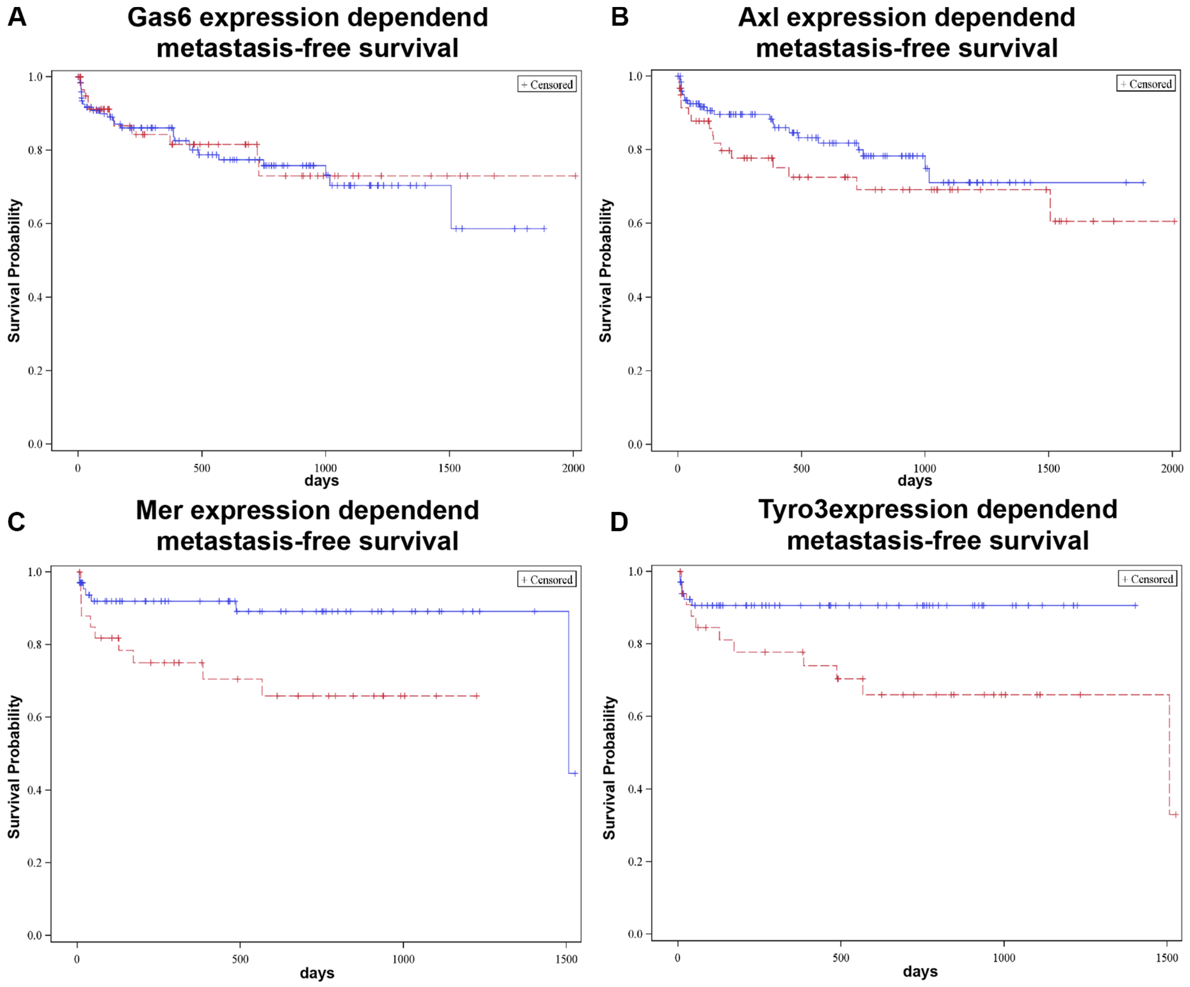


**Supplementary Figure S5: Gas6 and TAM receptor expression correlated to patients metastasis-free survival.** RT-PCR analysis was performed for Gas6, Axl, Mer and Tyro3 in colorectal tumor samples and normal mucosa of the patients listed in Table 1. The patient cohort was split in two groups comprising 33,3% of patients with the highest target gene expression (red) and the remaining 66,7% of patients (blue). Subsequently ΔΔCp values (Figure 1A, 1B and Supplementary Figure S1A, S1B) were correlated to patients metastasis-free survival. (**A**, **B**) Relative Gas6 and Axl mRNA expression is not associated with patients metastasis-free survival ($n$ = 200; $P$ = NS). (**C**, **D**) Relative Mer and Tyro3 mRNA expression in negative associated with patients metastasis-free survival ($n$ = 103; $P < .05$).

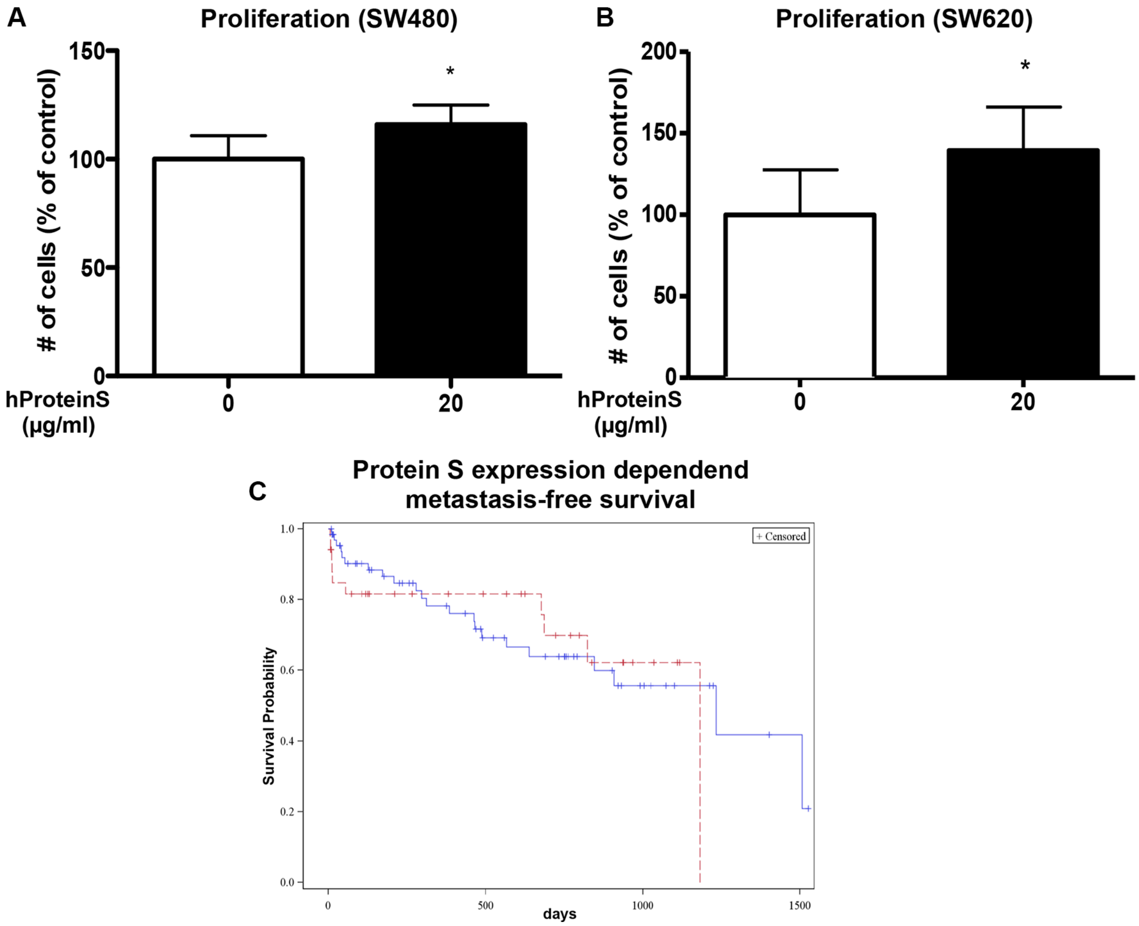


**Supplementary Figure S6: ProteinS induces proliferation *in vitro* but is not associated with patients survival *in vivo*.** (**A**, **B**) WST-1 reagent (Roche) was used to measure cell viability *in vitro*. Human ProteinS (hProteinS) induces proliferation of human colorectal cancer cell lines (SW480 and SW620) *in vitro* after 48 hours treatment ($n$ = 6; $P$ < .05). (**C**) RT-PCR analysis was performed for ProteinS in colorectal tumor samples and normal mucosa of the patients listed in Table 1. The patient cohort was split in two groups comprising 33,3% of patients with the highest target gene expression (red) and the remaining 66,7% of patients (blue). Subsequently ΔΔCp values were correlated to patients metastasis-free survival. Relative ProteinS mRNA expression is not associated with patients metastasis-free survival ($n$ = 103; $P$ = NS)

**Supplementary Table S1: Human primers for RT-PCR expression analysis in human tissue samples**

| Gene | Company | Sequence | Ref-No. |
|---|---|---|---|
| Gas6 | Qiagen | - | QT00049126 |
| Axl | Qiagen | - | QT00067725 |
| Mer | Qiagen | - | QT00031017 |
| Tyro3 | Qiagen | - | QT00055482 |
| Protein S | Qiagen | - | QT00011746 |
| 18s | Invitrogen | Fwd: AAA CGG CTA CCA CAT CCA AG<br>Rev: CCT CCA ATG GAT CCT CGT TA | - |

Primers were used for mRNA expression analysis in colorectal cancer tissue samples and normal mucosa as well as in colorectal liver metastasis and normal liver tissue respectively.

**Supplementary Table S2: Patient characteristics for mRNA expression analysis of Gas6, Axl, Mer,Tyro3 and ProteinS**

| | | *n* (%) or median (IQR) |
|---|---|---|
| | **Total *n*** | **200 (100)** |
| **Gender** | | |
| | male | 114 (57) |
| | female | 86 (43) |
| **Age (years)** | | 65,13 (27–88) |
| **Tumor size** | | |
| | T1 | 10 (5) |
| | T2 | 46 (23) |
| | T3 | 119 (59,5) |
| | T4 | 25 (12,5) |
| **Lymph node status** | | |
| | positive | 99 (49,5) |
| | negative | 101 (50,5) |
| **Distant metastases** | | |
| | positive | 55 (27,5) |
| | negative | 145 (72,5) |
| **UICC** | | |
| | I | 41 (20,5) |
| | II | 51 (25,5) |
| | III | 55 (27,5) |
| | IV | 53 (26,5) |
| **Tumor differentiation** | | |
| | high (G1) | 1 (0,5) |
| | moderate (G2) | 153 (76,5) |
| | poor (G3) | 44 (22) |
| | N.N. | 2 (1) |
| **Tumor location** | | |
| | colon | 101 (50,5) |
| | rectosigmoid | 13 (6,5) |
| | rectum | 86 (43) |
| **Neoadjuvant therapy** | | |
| | Yes | 46 (23) |
| | No | 151 (75,5) |
| | N.N. | 3 (1,5) |
| **Adjuvant therapy** | | |
| | Yes | 99 (49,5) |
| | No | 101 (50,5) |
| **Treatment** | | |
| | curative (R0) | 155 (77,5) |
| | palliative (R1/R2) | 45 (22,5) |

Summary of patient characteristics used for mRNA expression analysis of the target genes Gas6, Axl, Mer, Tyro3 and ProteinS in colorectal cancer tissue and normal mucosa of each patient respectively (*n* = 200).

## Supplementary Table S3: Patient characteristics for Gas6 and CD68 immunostainings

| | *n* (%) or median (IQR) |
|---|---|
| **Total *n*** | **102 (100)** |
| **Gender** | |
| male | 62 (60,8) |
| female | 40 (39,2) |
| **Age (years)** | 65 (21-88) |
| **Tumor size** | |
| T1 | 3 (2,9) |
| T2 | 21 (20,6) |
| T3 | 65 (63,7) |
| T4 | 13 (12,8) |
| **Lymph node status** | |
| positive | 47 (46,1) |
| negative | 55 (53,9) |
| **Distant metastases** | |
| positive | 22 (21,6) |
| negative | 80 (78,4) |
| **UICC** | |
| I | 20 (19,6) |
| II | 31 (30,4) |
| III | 28 (27,5) |
| IV | 23 (22,5) |
| **Tumor differentiation** | |
| High (G1) | 1 (1,0) |
| Moderate (G2) | 70 (68,6) |
| Poor (G3) | 30 (29,4) |
| N.N. | 1 (1,0) |
| **Tumor location** | |
| colon | 49 (48) |
| rectosigmoid | 6 (5,9) |
| rectum | 47 (46,1) |
| **Neoadjuvant therapy** | |
| Yes | 36 (35,3) |
| No | 66 (64,7) |
| **Adjuvant therapy** | |
| Yes | 41 (40,2) |
| No | 61 (59,8) |
| **Treatment** | |
| curative (R0) | 82 (80,4) |
| palliative (R1/R2) | 20 (19,6) |

Summary of patient characteristics used for immunohistochemical analysis of Gas6 and CD68 in human colorectal cancer tissue samples and normal mucosa of each patient respectively ($n$ = 102).

## Supplementary Table S4: Human and murine cell lines used for *in vitro* assays

| ATCC NO. | Name | Species | Origin | Histolgy | Tumor source | Mutant Gene | Mutation |
|---|---|---|---|---|---|---|---|
| CCL-222 | Colo205 | human | Colon | adenocarcinoma | Metastasis, Ascites | BRAF<br>KRAS<br>PIK3CA<br>TP53 | wt<br>wt<br>wt<br>mutant |
| CCL-221 | DLD-1 | human | Colon | adenocarcinoma | Primary tumor | BRAF<br>KRAS<br>PIK3CA<br>TP53 | wt<br>mutant<br>mutant<br>mutant |
| CCL-247 | HCT116 | human | Colon ascendens | carcinoma | Primary tumor | BRAF<br>KRAS<br>PIK3CA<br>TP53 | wt<br>wt<br>mutant<br>wt |
| HTB-38 | HT29 | human | Colon | carcinoma | Primary tumor | BRAF<br>KRAS<br>PIK3CA<br>TP53 | mutant<br>wt<br>mutant<br>mutant |
| CCL-228 | SW480 | human | Colon | adenocarcinoma | Primary tumor | BRAF<br>KRAS<br>PIK3CA<br>TP53 | wt<br>mutant<br>wt<br>mutant |
| CCL-227 | SW620 | human | Colon | adinocarcinoma | Lymph node metastasis | BRAF<br>KRAS<br>PIK3CA<br>TP53 | wt<br>mutant<br>wt<br>mutant |
| CRL-2638 | CT26 | mouse | Colon | carcinoma | - | BRAF<br>KRAS<br>PIK3CA<br>TP53 | wt<br>mutant<br>wt<br>wt |
| TIB-67 | J774A.1 | mouse | Monocyte, Macrophage | - | Ascites | - | - |

Detailed information about the different cell lines including mutations was collected from previously published literature (Ahmed D et al., Oncogenesis, 2013).

## Supplementary Table S5: Human and murine primers for RT-PCR expression analysis *in vitro*

| Gene | Species | Company | Sequence | Ref-No. |
|---|---|---|---|---|
| Gas6 | human | Qiagen | - | QT00049126 |
| Gas6 | mouse | Qiagen | - | QT00101332 |
| Axl | human | Qiagen | - | QT00067725 |
| Axl | mouse | Qiagen | - | QT00101353 |
| Tyro3 | human | Qiagen | - | QT00055482 |
| Tyro3 | mouse | Qiagen | - | QT00197659 |
| Mer | human | Qiagen | - | QT00031017 |
| Mer | mouse | Qiagen | - | QT00148561 |
| 18s | human | Invitrogen | Fwd: AAA CGG CTA CCA CAT CCA AG<br>Rev: CCT CCA ATG GAT CCT CGT TA | - |
| 18s | mouse | Invitrogen | Fwd: GTA ACC CGT TGA ACC CCA TT<br>Rev: CCA TCC AAT CGG TAG TAG CG | - |

Primers were used for baseline mRNA expression analysis of Gas6, Axl, Mer and Tyro3 as well as after 5-FU treatment *in vitro*.

## Supplementary Table S6: Murine primers for RT-PCR expression analysis of M1 and M2 phenotype macrophages *in vitro*

| Gene | Company | Sequence | Ref-No. |
|---|---|---|---|
| iNOS | Invitrogen | Fwd: CTC ACT GGG ACA GCA CAG AA<br>Rev: GGT CAA ACT CTT GGG GTT CA | - |
| IL-6 | Invitrogen | Fwd: CAA AGC CAG AGT CCT TCA GAG<br>Rev: GCC ACT CCT TCT GTG ACT CC | - |
| Arginase | Invitrogen | Fwd: GTG TAC ATT GGC TTG CGA GA<br>Rev: AGG TGA ATC GGC CTT TTC TT | - |
| CCR2 | Invitrogen | Fwd: CCT GCA AAG ACC AGA AGA GG<br>Rev: TAT GCC GTG GAT GAA CTG AG | - |

Primers of M1 (iNOS, IL-6) and M2 (Arginase, CCR2) phenotype specific genes were selected to verify the differentiation of J774A.1 towards an M1 or M2 phenotype by LPS (1 µg/ml) or M-CSF (10 ng/ml) treatment respectively.

## Supplementary Table S7: Human siRNAs for TAM receptor knockdown *in vitro*

| Gene | Company | Sequence | Ref-No. |
|---|---|---|---|
| Axl | Invitrogen | Fwd: CCA GGA ACU GCA UGC UGA AUG AGA A<br>Rev: UUC UCA UUC AGC AUG CAG UUC CUG G | HSS183343 |
| Mer | Invitrogen | Fwd: CCA GAA CCA UGA GAU GUA UGA CUG U<br>Rev: AUA GUC AUA CAU CUC AUG GUU CUG G | HSS116030 |
| Tyro3 | Invitrogen | Fwd: GCU GUG CCU CCA AAC UGC CUG UCA A<br>Rev: UUG ACA GGC AGU UUG GAG GCA CAG C | HSS187439 |

Human colorectal cancer cell line HCT116 was transfected with TAM receptor siRNAs and proliferation was assessed *in vitro* using the Cell Proliferation Reagent WST-1 (Roche).
